# PHYSICAL CONDITIONS ASSOCIATED WITH SUICIDE ATTEMPTS: DO RISKS DIFFER AMONG OUR OLDER VETERANS?

**DOI:** 10.1093/geroni/igz038.3038

**Published:** 2019-11-08

**Authors:** Emily S Bower, Kelsey Simons, Patrick G Walsh, Lisham Ashrafioun, Kimberly A Van Orden, Todd M Bishop

**Affiliations:** 1 VISN Center of Excellence for Suicide Prevention, Canandaigua, New York, United States; 2 University of Rochester School of Medicine, Rochester, New York, United States

## Abstract

Physical illness confers risk for late-life suicide, yet few studies report whether risks differ with older age and among Veterans. We examined age-stratified associations between physical illness and suicide attempt among Veterans 65+ years (n=8452, 97% male) from a larger retrospective case-control study that utilized secondary data from the Veterans Affairs Corporate Data Warehouse and Suicide Prevention Applications Network. Controls were matched by age, sex, and mental health treatment utilization. Risk estimates for 15 conditions and a combined comorbidity score were stratified by young-old (65-74), middle-old (75-84), and oldest-old (85+), adjusting for age and sex within strata. Neurodegenerative disorder (ORs=4.5, 6.0, 6.5) or dementia (ORs=5.0, 5.7, 4.4) diagnosis within 180 days conferred the highest risks across young-, middle-, and oldest-old. Cerebrovascular disorder was associated with higher risk among the oldest-old versus young-old (ORs=6.1 vs 2.2). Findings differ from reported risks for suicide death. Illness may be experienced differently across later-life.

